# Panton–Valentine Leukocidin Colocalizes with Retinal Ganglion and Amacrine Cells and Activates Glial Reactions and Microglial Apoptosis

**DOI:** 10.1038/s41598-018-20590-z

**Published:** 2018-02-13

**Authors:** XuanLi Liu, Pauline Heitz, Michel Roux, Daniel Keller, Tristan Bourcier, Arnaud Sauer, Gilles Prévost, David Gaucher

**Affiliations:** 1 Université de Strasbourg, Hôpitaux Universitaires de Strasbourg, Fédération de Médecine Translationnelle de Strasbourg, EA7290 Virulence Bactérienne Précoce, Institut de Bactériologie, Strasbourg, France; 2 Hôpitaux Universitaires de Strasbourg, Service d’Ophtalmologie du Nouvel Hôpital Civil, Strasbourg BP426, 67091 cedex, France; 3 Department of Translational Medicine and Neurogenetics, Institut de Génétique et de Biologie Moléculaire et Cellulaire, CNRS UMR_7104, Inserm U 964, Université de Strasbourg, Illkirch, France

## Abstract

Experimental models have established Panton–Valentine leukocidin (PVL) as a potential critical virulence factor during *Staphylococcus aureus* endophthalmitis. In the present study, we aimed to identify retinal cell targets for PVL and to analyze early retinal changes during infection. After the intravitreous injection of PVL, adult rabbits were euthanized at different time points (30 min, 1, 2, 4 and 8 h). PVL location in the retina, expression of its binding receptor C5a receptor (C5aR), and changes in Müller and microglial cells were analyzed using immunohistochemistry, Western blotting and RT-qPCR. In this model of PVL eye intoxication, only retinal ganglion cells (RGCs) expressed C5aR, and PVL was identified on the surface of two kinds of retinal neural cells. PVL-linked fluorescence increased in RGCs over time, reaching 98% of all RGCs 2 h after PVL injection. However, displaced amacrine cells (DACs) transiently colocalized with PVL. Müller and microglial cells were increasingly activated after injection over time. IL-6 expression in retina increased and some microglial cells underwent apoptosis 4 h and 8 h after PVL infection, probably because of abnormal nitrotyrosine production in the retina.

## Introduction

Bacterial endophthalmitis is a common but severe infection of the eye, which is often caused by ocular surgery or trauma^1^. The visual prognosis of endophthalmitis depends on many factors, one of the most important being the virulence of the infecting bacteria, as reported in a recent study^2^. Though *Staphylococcus aureus* is rarely involved in ocular endophthalmitis, it secretes an extensive repertoire of cytotoxins that represent a significant threat to visual outcomes.

Methicillin-resistant *S*. *aureus* (MRSA) places a significant burden on healthcare resources due to its resistance to antimicrobial treatment. Community-associated (CA)-MRSA, which emerged in 1990s, can be distinguished from hospital-acquired (HC)-MRSA, primarily because it bears the gene encoding Panton–Valentine leukocidin (PVL)^3,4^. Recent studies have reported that the percentage of CA-MRSA has significantly increased in MRSA infection isolates and may continue to rise in the future due to the horizontal transfer of genes and inter-human transmission^5^. PVL is composed of two distinct proteins, a class S (31–32 kDa) and a class F component (33–34 kDa), which organize as alternate octamers, called prepores, and are internalized into polymorphonuclear cells where they initiate intracellular relapsing of Ca^2+^ storages. The class S component binds to the C5a membrane receptor (C5aR), allowing the secondary interaction of the F component. Unaccompanied class S or F proteins never seem to produce any effect on targeted cells^6^. PVL may result in tissue necrosis during *S*. *aureus* infection, especially necrotizing infections, such as furuncles, acute necrotizing pneumonia, and osteomyelitis^7,8^.

Recent studies have reported that PVL binds to human complement C5a receptor. This binding largely decreases in rodents but is conserved in rabbits^9^. Recent studies on humanized mice with C5aR showed the critical role played by PVL in the determination of necrotizing pneumonia and its severity^10,11^. Other staphylococcal leukotoxins have been characterized, but only LukS-PV and HlgC were shown to bind C5aR^10,11^. There has been evidence of PVL targeting myeloid cells such as monocytes (M), macrophages (Mϕ), and polymorphonuclear cells (PMNs), but not lymphocytes^12^. Another recent study showed that *in vitro*, PVL could target dorsal root neurons and cerebellum granular neurons^13^. In the rabbit eye, previous studies revealed that PVL and other staphylococcal leukotoxins injected into the rabbit vitreous could cause retinal inflammation and breakdown of the blood–retinal barrier (BRB)^14,15^. However, the mechanism leading to such inflammation remains to be determined albeit vitreous is mainly devoid of cells.

IL-6 has been shown to have effective angiogenic activities and inflammatory role in models of choroidal neovascularization, ocular inflammation and tumor angiogenesis^16^. In this study, our purpose was to identify retinal cell targets for PVL and to analyze initial mechanisms that might support or indicate inflammation and inter-cellular communication. In particular, we studied the potential consequences of retinal cell infection, such as glial reaction, neuronal cell damage, and the presence of inflammatory markers. The results reported herein strongly suggest that PVL colocalized with two types of retinal neurons: displaced amacrine cells (DACs) very early in the translocation process, and retinal ganglion cells (RGCs), which triggered a glial reaction and an increase of IL-6 expression in the retina.

## Results

### PVL was located in RGCs and DACs

An anti-LukS-PV antibody (Table 1) was used to identify PVL translocation from the rabbit vitreous into the retina. Results indicated that PVL was concentrated in the ganglion cell layer. Since this cell layer is composed of RGCs and DACs, we investigated the exact target of PVL using specific labeling for RGCs (anti-RBPMS antibody) and DACs (anti-CHAT antibody) (Table 1).

**Table 1:** list of Specific markers used in the current study.

Primary antibodies or lectin			
Target	Antiserum	Source	Concentration
PVL	Rabbit anti-LukS-PV polyclonal	EA-7290, Strasbourg, France	2 µg/mL
C5aR	Rabbit anti-C5aR polyclonal	Abcam, Cambridge, UK	2 µg/mL
Ganglion cells	Guinea pig anti-RBPMS polyclonal	UCLA Neurobiology, Los Angeles, CA, USA	2 µg/mL
Displaced amacrine cells	Goat anti-CHAT polyclonal	Chemicon Merck-Millipore, Temecula, CA, USA	20 µg/mL
Müller cells	Mouse anti-GFAP polyclonal	Bio-Rad AbD Serotec, Oxfordshire, UK	2 µg/mL
Microglial cells	Cy3-tagged GSAI-B4	Sigma Aldrich, Saint Louis, MO, USA	2 µg/mL
Nitrotyrosine	Mouse anti-nitrotyrosine monoclonal	Santa Cruz Biotechnology, Heidelberg, Germany	2 µg/mL
C5L2	Rabbit anti-C5L2 polyclonal	GeneTex, San Antonio, TX, USA	2 µg/mL
β-actin	Rabbit anti-β-actin polyclonal	Santa Cruz Biotechnology, Heidelberg, Germany	1:2000
IL-6	Mouse anti-IL-6 monoclonal	Abbexa Ltd, Cambridge, UK	1:2000
IL-8	Mouse anti IL-8 monoclonal	Abbexa Ltd, Cambridge, UK	1:2000
IL-1β	Rabbit anti-IL-1β polyclonal	Abbexa Ltd, Cambridge, UK	1:2000
TNF-α	Mouse anti-TNFα monoclonal	Abbexa Ltd, Cambridge, UK	1:2000
**Secondary antibodies**			
Anti-rabbit	Goat and donkey polyclonal Alexa 555 nm-conjugated	Life Technologies, Carlsbad, CA, USA	2 µg/mL
Anti-goat	Donkey polyclonal Alexa 488-conjugated	Molecular Probes, Eugene, OR, USA	2 µg/mL
Anti-mouse	Donkey polyclonal Alexa 488-conjugated	Abcam	2 µg/mL
Anti-guinea pig	Goat polyclonal Alexa 488-conjugated	Abcam	2 µg/mL
TUNEL	DNA strand breaks	Roche Life Science, Indianapolis, IN, USA	—
Nuclei	Hoechst 33258	Molecular Probes^TM^, Eugene, OR, USA	0.1 µg/mL
Anti-rabbit	Anti-Rabbit IgG (whole molecule)–Peroxidase	Sigma Aldrich, Saint Louis, MO, USA	1:10000
Anti-mouse	Goat anti-mouse IgG-Peroxidase	Santa Cruz Biotechnology, Heidelberg, Germany	1:10000

RBPM, RNA-binding protein with multiple splicing; CHAT, choline acetyl transferase; GFAP, glial fibrillary acidic protein; GSAI, *Griffonia simplicifolia* agglutinin isolectin. TUNEL, terminal deoxynucleotidyl transferase dUTP nick-end labelling.

**Figure 1:**
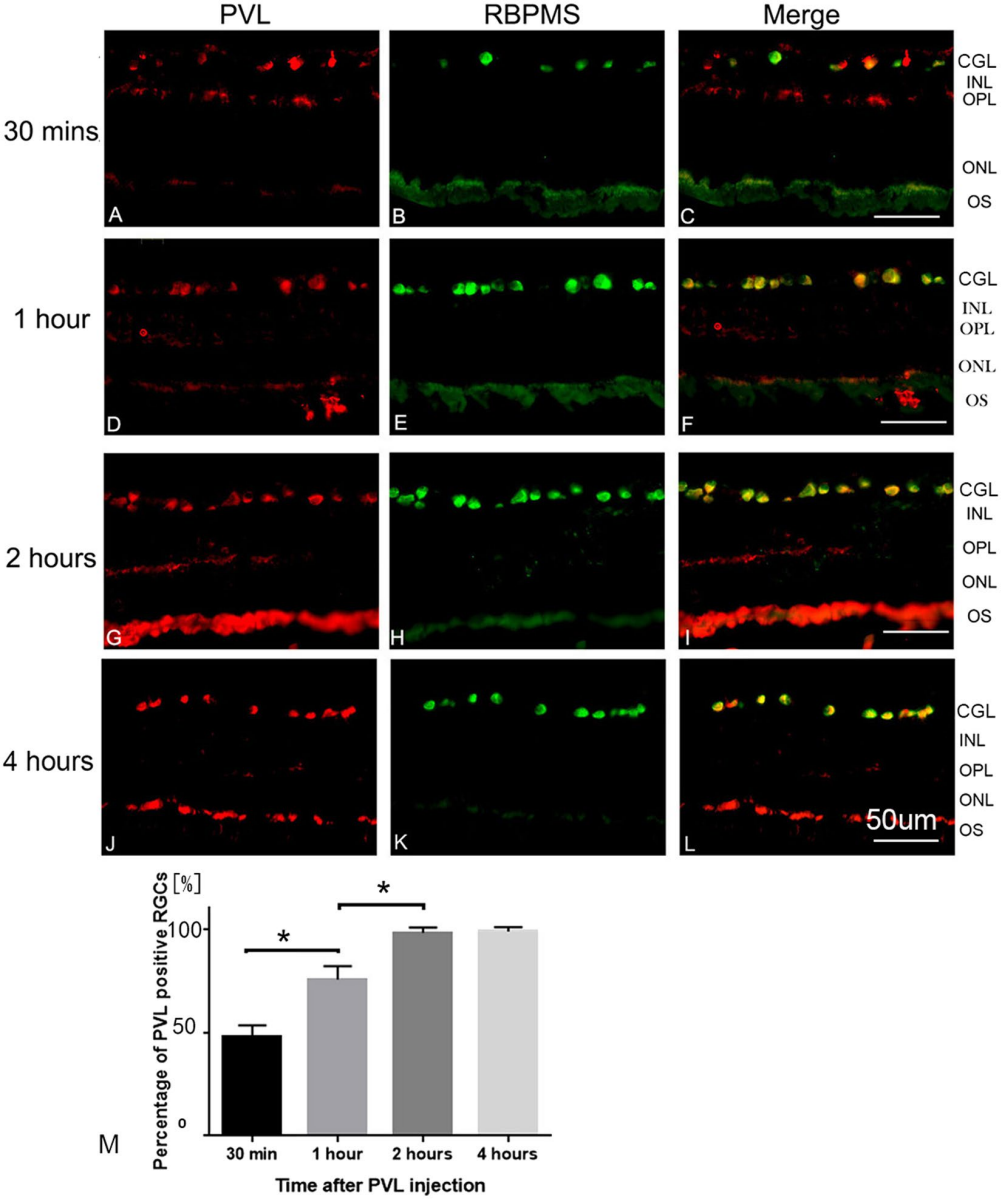
PVL expression in RGCs. PVL (red fluorescence **A**,**D**,**G**,**J**) colocalized with RGCs labeled with anti-RBPMS antibody (green fluorescence **B**,**E**,**H**,**K**) in the retinal vertical sections 30 min (**A**–**C**), 1 h (**D**–**F**), 2 h (**G**–**I**), and 4 h (**J**–**L**) after PVL injection. The number of PVL-positive RGCs increased with time (**C**,**F**,**I**,**L** yellow fluorescence), the rate of PVL-positive RGCs were 47%, 76%, 98%, 99% for 30 min and 1, 2, and 4 h (M, ***p < 0.001, *p < 0.05 *n* = 3 eyes at each time point). Abbreviated symbols: PVL, Panton–Valentine leukocidin; RGCs, retinal ganglion cells; RBPMS, RNA-binding protein with multiple splicing; GCL, ganglion cell layer; INL, inner nuclear layer; OPL, outer plexiform layer; ONL, outer nuclear layer; OS, photoreceptor outer segments.

**Figure 2:**
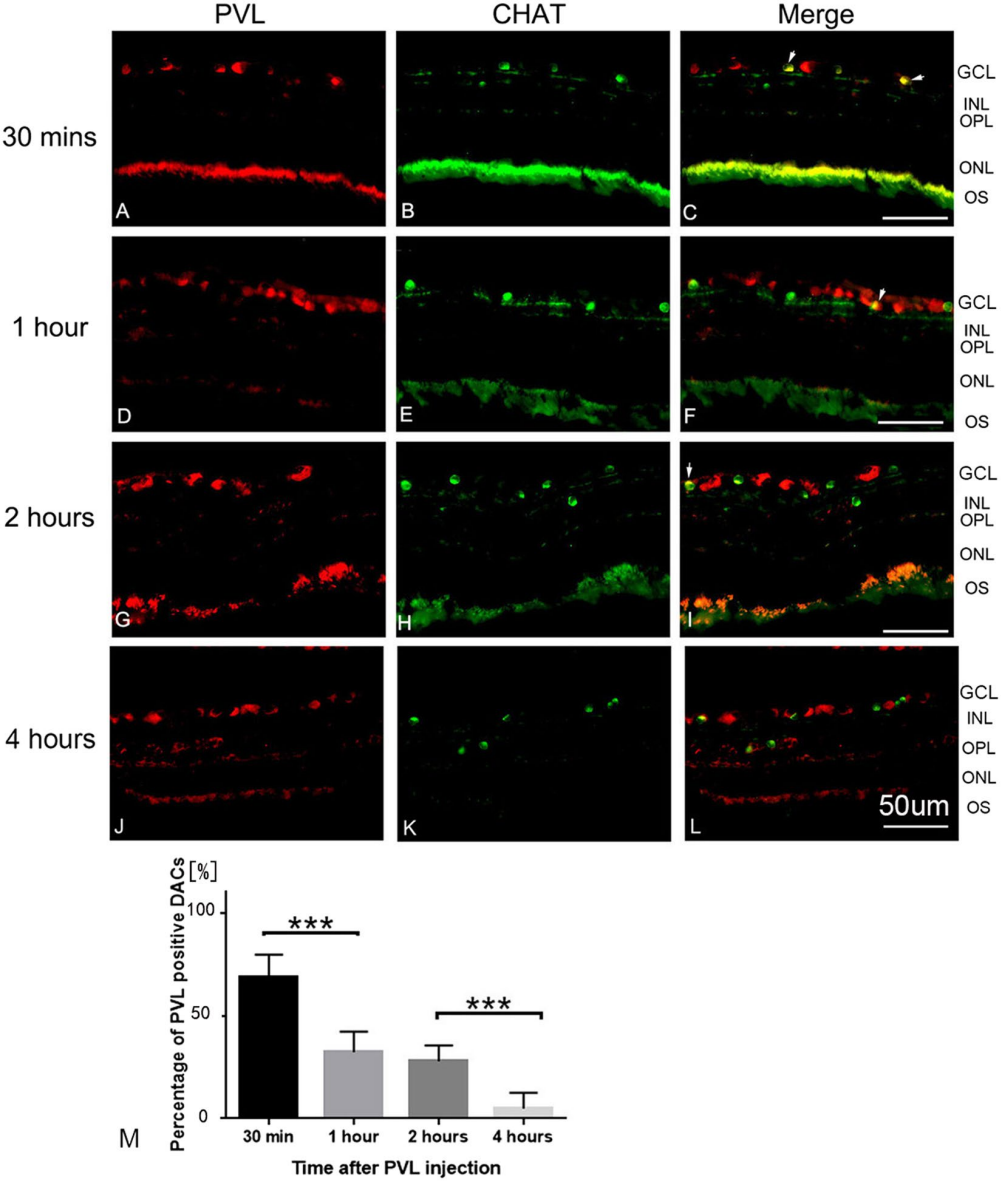
PVL expression was transient in DACs. Thirty minutes after PVL injection, the majority of DACs labeled with anti-CHAT antibody (green fluorescence **B**,**C**) colocalized with PVL (red fluorescence **A**,**C**). After 1 h, the PVL-positive DACs decreased (**D**–**L**). The percentage of PVL-positive DACs were 68%, 32%, 27%, 5% for 30 min and 1, 2, and 4 h (M, ***p < 0.001, *p < 0.05, *n* = 3 eyes at each time point). Abbreviated symbols: PVL, Panton–Valentine leukocidin; DACs, displaced amacrine cells; CHAT, choline acetyl transferase; GCL, ganglion cell layer; INL, inner nuclear layer; OPL, outer plexiform layer; ONL, outer nuclear layer; OS, photoreceptor outer segments.

RGCs were PVL-positive. The rate of positive RGCs significantly increased from 47% to 76% from 30 min to 1 h (*p* < 0.05) after PVL injection. This rate reached 98% after 2 h (*p* < 0.05 compared with the rate of 1 h) and 99% 4 h after PVL injection (Fig. 1). The majority of DACs were PVL-positive at 30 min after PVL injection. However, the rate of PVL-positive DACs significantly decreased from 68% to 32% between 30 min and 1 h (*p* < 0.01) after PVL injection, respectively. This rate continued to significantly decrease and was 27% at 2 h and 5% at 4 h following PVL injection (*p* < 0.01) (Fig. 2). Eight hours after PVL injection, all RGCs colocalized with PVL, while 4% of DACs still colocalized with PVL (see Supplementary Fig. S1).

**Figure 3:**
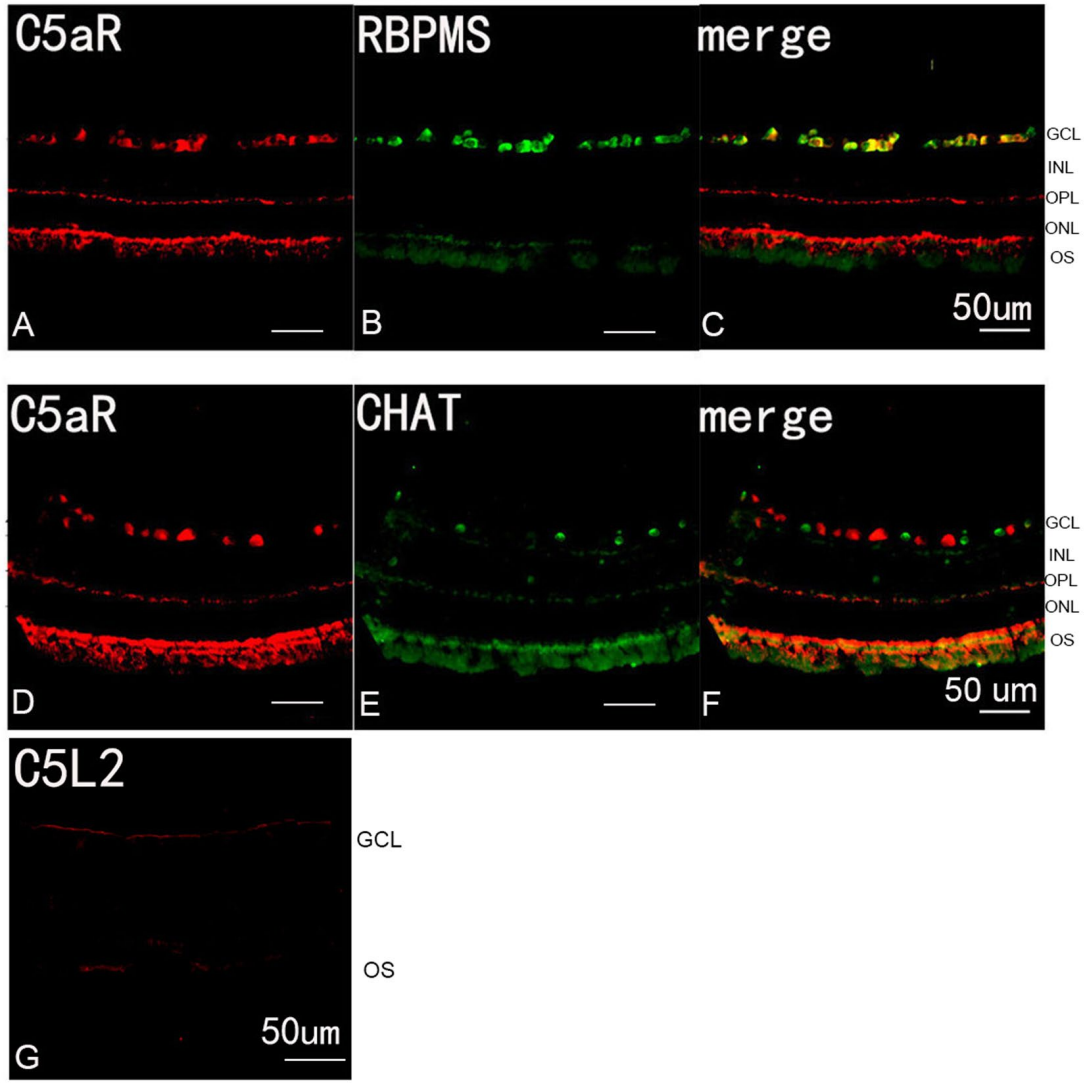
C5aR was expressed by RGCs. Double-labeling immunohistochemistry in the control eyes showed that RGCs labeled with anti-RBPMS antibody (green fluorescence **A**,**C**) colocalized with anti-C5aR staining (red fluorescence **B**,**C**), and the DACs labeled with anti-CHAT antibody (green fluorescence **E**,**F**) did not colocalize with anti-C5aR staining (red fluorescence **D**,**F**). The anti-C5L2 labeling did not show specific staining in retina (red fluorescence **G**). Abbreviated symbols: RGCs, retinal ganglion cells; RBPMS, RNA-binding protein with multiple splicing; DACs, displaced amacrine cells; CHAT, choline acetyl transferase; GCL, ganglion cell layer; INL, inner nuclear layer; OPL, outer plexiform layer; ONL, outer nuclear layer; OS, photoreceptor outer segments.

To further investigate whether PVL was specifically colocalized with the RGCs and DACs, we examined the rabbit retina for C5aR and C5L2 expression using specific antibodies, since PVL binds with human neutrophils through the C5a receptor^9,17^. The anti-C5L2 antibody did not detect any specific staining in rabbit retina (Fig. 3G). C5aR was consistently expressed in RGCs suggesting that PVL might colocalize with RGCs through C5aR. C5aR did not colocalize with DACs (Fig. 3 and Table 1). Therefore, another mechanism for PVL colocalization (possibly with a decreased affinity) and possible penetration in DACs cannot be excluded.

### The Müller and microglial cells were activated after PVL injection

**Figure 4:**
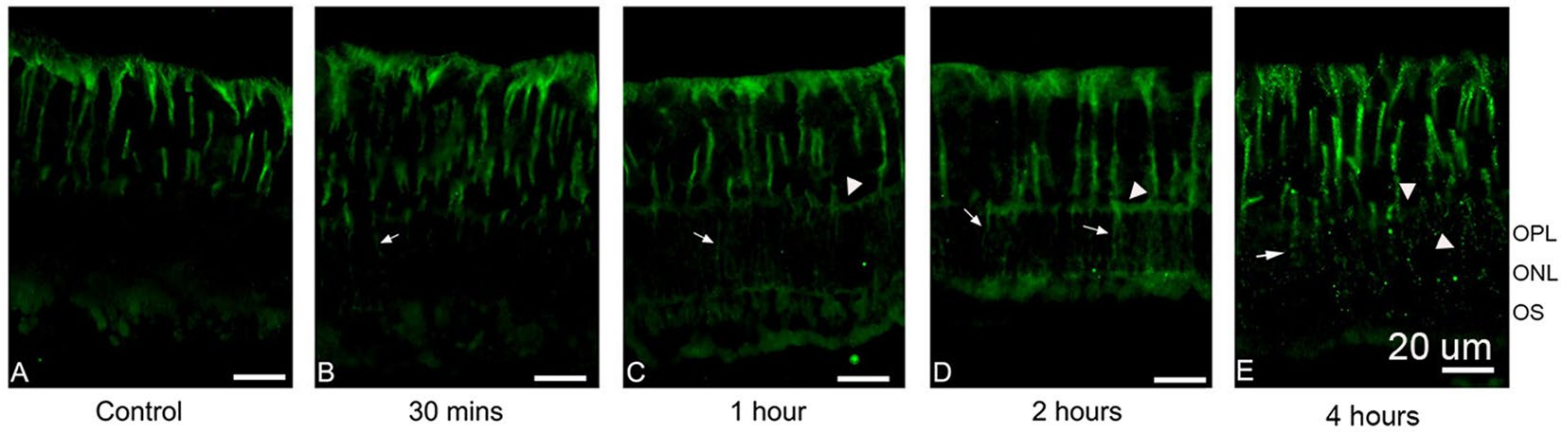
Müller cell reactivity was observed as early as 30 min after PVL injection. Compared with control eyes (green fluorescence **A**), anti-GFAP labeling was abnormally present in outer retina in PVL-injected eyes (green fluorescence **B**–**E**). More anti-GFAP-stained Müller processes (arrows) were noted at 1 h (**C**), 2 h (**D**), and 4 h (**E**) than at 30 min after PVL injection. At 1 h and 2 h, OPL was well defined with GFAP staining (arrowheads). At 4 h, disruptions of GFAP staining within outer processes and the OPL were noticed (arrowheads). (*n* = 3 eyes at each time point). Abbreviated symbols: GFAP, glial fibrillary acidic protein; OPL, outer plexiform layer; ONL, outer nuclear layer; OS, photoreceptor outer segments.

Müller cells can transform into an activated state, characterized by the rapid upregulation of glial fibrillary acidic protein (GFAP). This upregulation occurs after acute retinal injuries or inflammation states^18^. Anti-GFAP antibody was used to label Müller cells (Table 1). As early as 30 min after PVL injection, Müller cells abnormally expressed GFAP in the outer retina (Fig. 4 and Table 1). This abnormal GFAP expression was not observed in the controls. Only a few Müller cell processes stained with GFAP were visible in the outer retina at 30 min, whereas the number and extension of processes increased from 1 to 2 h after PVL injection. At these time points, the outer plexiform layer (OPL) was well defined with GFAP staining. At 4 h, it seemed that the architecture of the retina had changed as numerous disruptions of GFAP staining within outer processes and the OPL were noticed (Fig. 4). These disruptions were not detected at earlier time points (Fig. 4).

**Figure 5:**
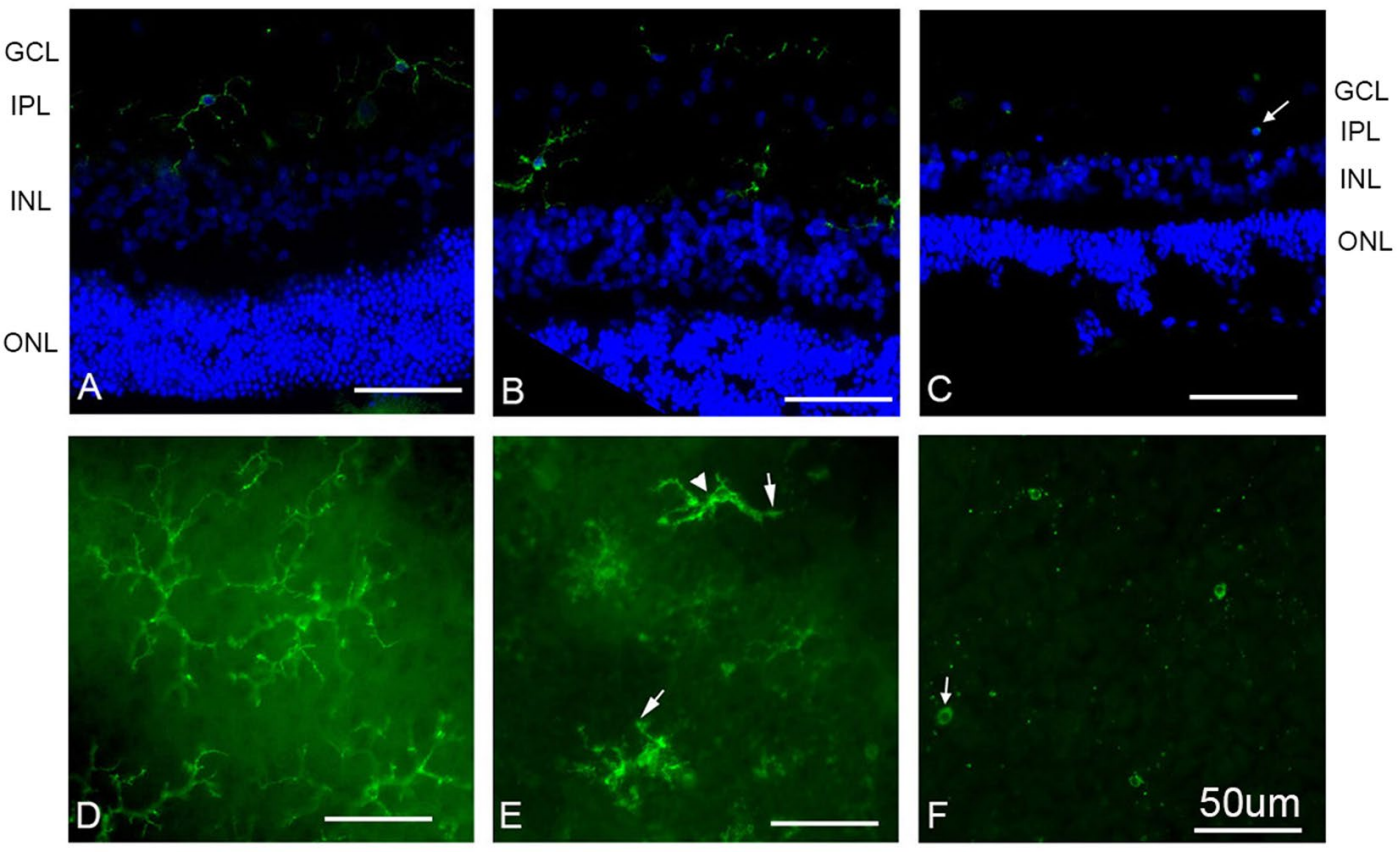
The microglial cells underwent morphological changes 2 h after PVL injection. (**A**–**C**) were vertical retinal sections, while (**D**–**F**) were whole retinal mounts. Cy3-tagged GSAI-B4 labeled microglial cell. Hoechst stained nuclei (blue fluorescence **A-C**). No microglial cell migration was observed (green fluorescence **A**–**C**). However, microglial cells showed retracted dendrites (arrow) and enlarged proximal parts of processes (arrowhead) (green fluorescence **B**,**E**) compared to controls (green fluorescence **A**,**D**). After 4 h, the microglial processes disappeared (arrow) (green fluorescence **C**,**F**). (*n* = 3 eyes at each time point). Abbreviated symbols: GCL, ganglion cell layer; IPL, inner plexiform layer; INL, inner nuclear layer; ONL, outer nuclear layer.

Morphological changes of Cy3-tagged GSAI-B4 labeled microglial cells (Table 1) were observed 2 h following PVL injection. The cell bodies and dendrites were enlarged, and the number of dendritic processes clearly decreased, which may correspond to an early activation state^19^. After 4 h, the microglial dendritic processes disappeared. However, no apparent microglial cell migration across the retina was observed on vertical sections (Fig. 5).

### Some retinal microglial cells underwent apoptosis

**Figure 6:**
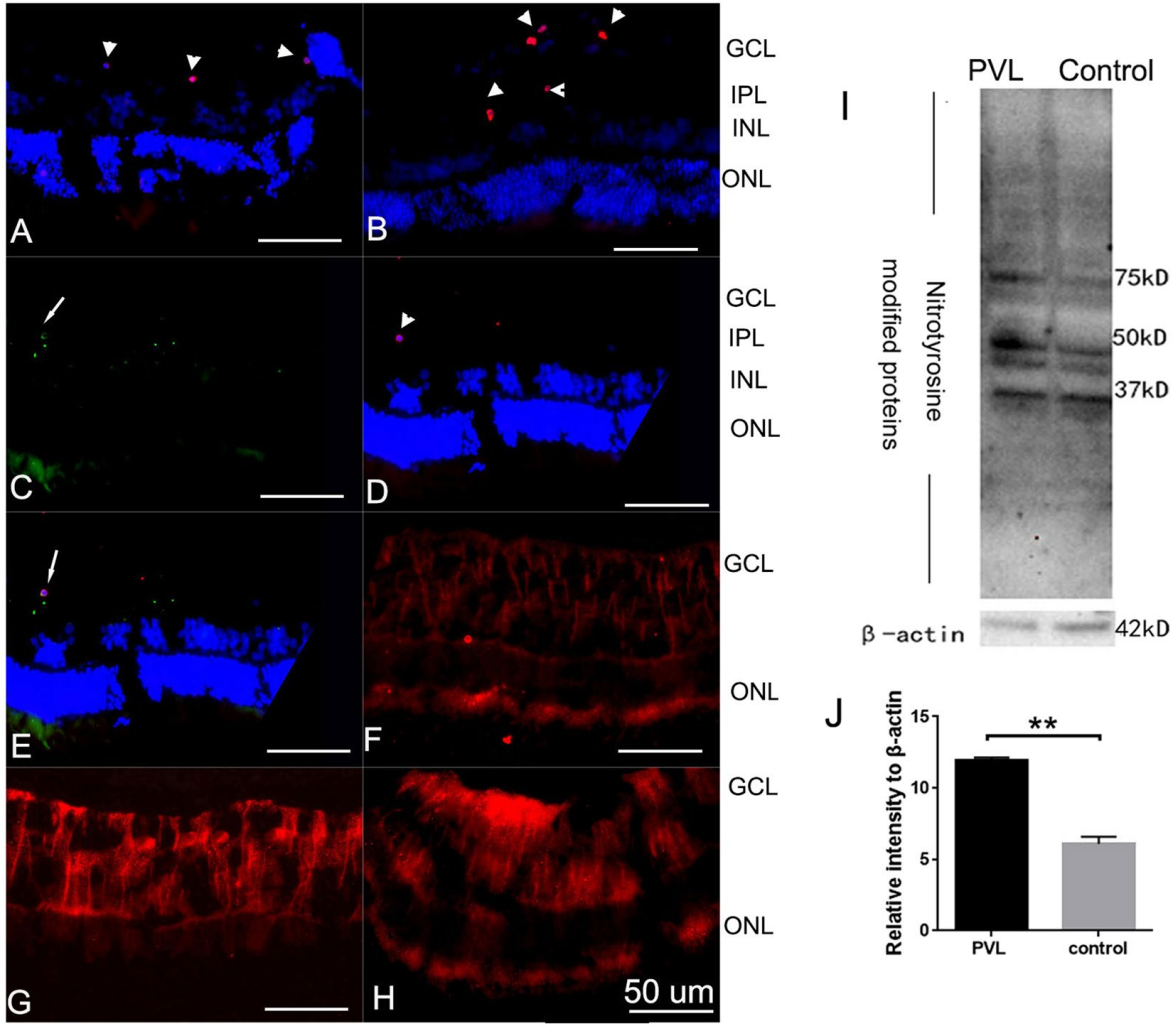
Some microglial cells underwent apoptosis, nitrotyrosine accumulated in the retina 4 h and 8 h after PVL injection. Apoptotic cells were situated in inner retina at 4 h (arrow head, red fluorescence **A**) and 8 h (arrow head, red fluorescence **B**) after PVL injection. The TUNEL-positive cells (arrow head, red fluorescence **D**,**E**) colocalized with microglial cells (arrow, green fluorescence **C**,**E**). Hoechst stained nuclei (blue fluorescence **A**,**B**,**D**,**E**). RGCs and DACs did not colocalize with TUNEL-positive cells (Supplementary Fig. S3). The immune activity of nitrotyrosine increased in the retina 4 h (red fluorescence G) and 8 h (red fluorescence **H**) after PVL injection compared with controls (red fluorescence **F**). Western blotting experiments showed that nitrotyrosine-modified proteins were expressed (migration of bands between 37 to 75 kDa) in both PVL-treated 4 h retinas and controls. The full-length blots are presented in Supplementary Fig. S5. Nitrotyrosine and β-acitin blot were from the same samples in the same gel (**I**). However, the total intensity of bands quantified by densitometry was increased almost two times in PVL-treated 4 h retinas compared to controls. (**I**,**J**). (**p < 0.01, *n* = 3 eyes). Abbreviated symbols: TUNEL, terminal deoxynucleotidyl transferase dUTP nick-end labeling; RGCs, retinal ganglion cells; DACs, displaced amacrine cells. GCL, ganglion cell layer; IPL, inner plexiform layer; INL, inner nuclear layer; ONL, outer nuclear layer; OS, photoreceptor outer segments. ns: no significant difference.

Terminal deoxynucleotidyl transferase dUTP nick-end labeling (TUNEL)-positive cells were located in the inner plexiform layer and ganglion cell layer 4 h and 8 h after PVL injection (Fig. 6**A**,**B**), while TUNEL were negative in control retinas and PVL injected retinas at 30 min, 1 h, 2 h time points retinas (see Supplementary Fig. S2). TUNEL-positive cells did not colocalize with either RBPMS-immunoreactive RGCs or CHAT-immunoreactive DACs (see Supplementary Fig. S3 and Table 1). Only microglial cells colocalized with TUNEL-positive cells, as shown in Fig. 6. At 4 h, the mean number of TUNEL-positive cells/field was 1.06. At 8 h, the number of apoptotic cells increased: mean number of TUNEL-positive cells/field was 1.86.

### IL-6 and nitrotyrosine inflammation markers increased 4 h and 8 h after PVL injection

The immunohistochemistry staining of nitrotyrosine 4 h and 8 h after PVL injection showed increased nitrotyrosine accumulation in the retina compared with the controls (Fig. 6**F**,**G**,**H**). The nitrotyrosine-modified proteins increased by almost two times in PVL-treated retinas after 4 h compared with the controls, as demonstrated by Western blotting (Fig. 6**I**,**J**). Nitrotyrosine is a stable marker of peroxynitrite-mediated oxidative damage, which is indicative of nitric oxide (NO) production in the retina after PVL injection.

**Figure 7:**
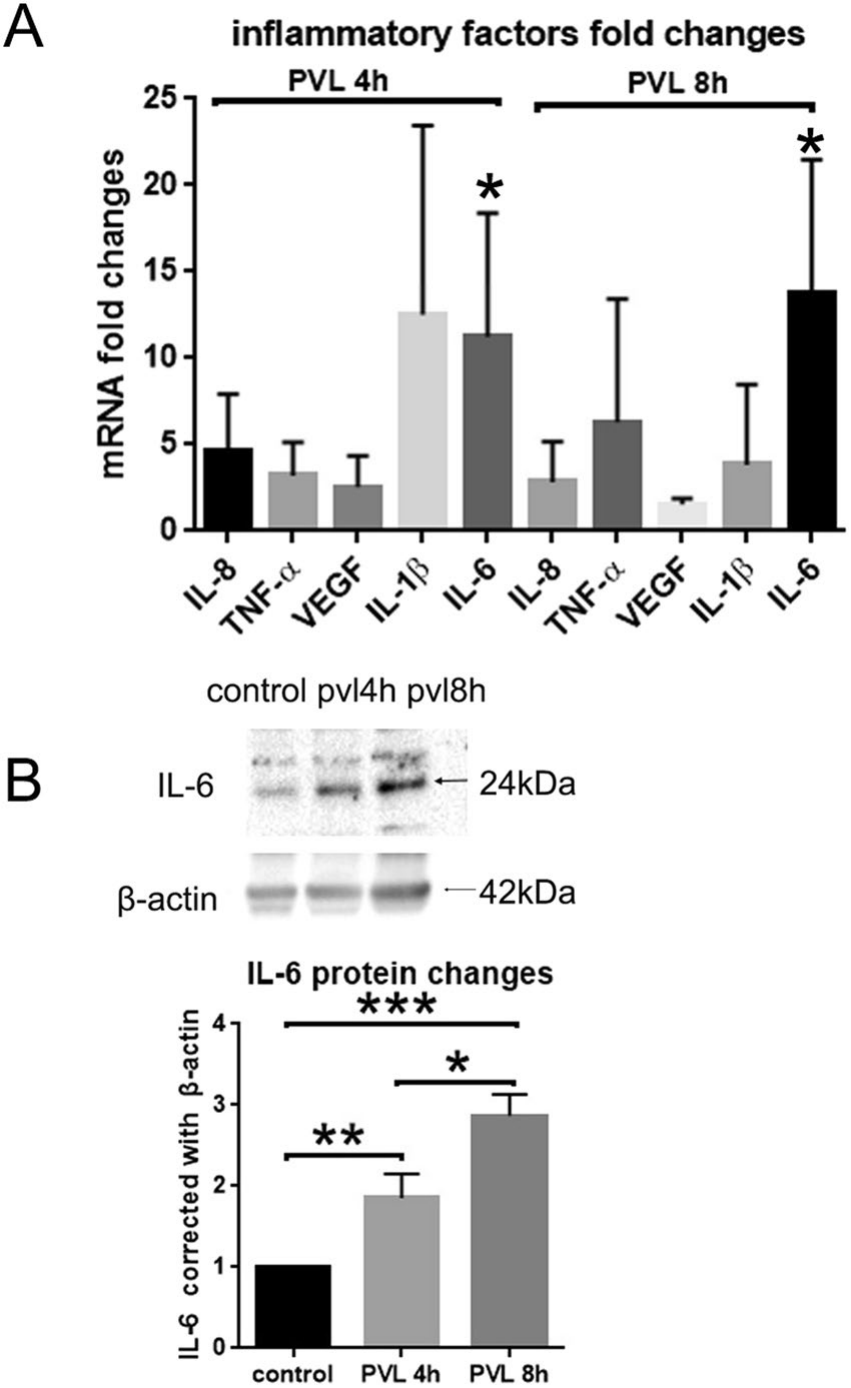
IL-6 mRNA and protein expression were increased 4 h and 8 h after PVL injection. The mRNA extracted from retinal tissue were analyzed by RT-qPCR. The mRNA (IL-8, TNF-α, VEGF, IL-1β, IL-6) levels were normalized by those of β-actin and presented as fold changes relative to control groups. The results ∆Ct were analyzed statistically by the paired t-test. Only IL-6 showed significant difference, increasing 11.24 ± 4.123 folds at 4 h and 13.74 ± 4.457 folds at 8 h after PVL injection (**A**, *p < 0.05, n = 3 eyes for each group). The proteins expression of IL-8, TNF-α, IL-1β, IL-6 were also semi-quantitatively measured by Western blotting. The full-length blots are presented in Supplementary Figs S4, 6, 7. Only IL-6 expression significantly increased by 1.85 ± 0.3 folds at 4 h and 2.87 ± 0.26 folds at 8 h after PVL injection. The blots of IL-6 and β-actin were from the same samples in the same gels with different exposure time (**B**, *p < 0.05, **p < 0.01, ***p < 0.005 n = 3 eyes for each group).

RT-qPCR test revealed that IL-6 mRNA significantly increased in retinal tissue by 11.24 folds and 13.74 folds at 4 and 8 h respectively after PVL injection compared to controls (Fig. 7**A**). Semi-quantitative analysis of western blot results showed that IL-6 proteins expression in retinal tissue was also increased by 1.85 folds at 4 h and 2.87 folds at 8 h after PVL injection compared to controls (Fig. 7**B**). mRNA and protein expression of other inflammatory factors such as IL-8, TNFα, IL-1β, VEGF MCP-1 were also measured by RT-qPCR and western blot. But no significant difference, albeit variations, were noted between controls and PVL infected retinas (Fig. 7**A** and Supplementary Fig. S4).

## Discussion

PVL colocalizes with RGCs through C5aR and transiently colocalizes with DACs through an unknown mechanism in the rabbit retina. Müller and microglial cells are activated after that PVL colocalizes with both RGC and DAC neural cells.

The exact mechanism by which neural cells are activated remains unknown. A recent report established that LukS-PV can bind C5aR and C5L2, the two complement C5a receptors, to mediate the toxin binding and toxicity in rabbit and human blood cells^9^. C5a receptors are abundantly expressed in myeloid cells, but they are less expressed in non-myeloid cells. Neural cells have been identified to express functional complement C5a receptors^20^. In general, we consider that C5aR is the major receptor and C5L2 is the minor receptor for C5a, and possibly for PVL. C5L2 is an intracytoplasmic G-protein-coupled receptor, never present at the cell membrane. C5L2 does not couple with G proteins, is not found to have a direct signaling function^21^, and is not identified in any neurons. In addition, the quantity of mRNA and protein of C5L2 are significantly lower than those of C5aR in leukocytes, although C5L2 has nearly similar affinity to LukS-PV as C5aR in U937 C5aR-transfected cells^22^. The findings of the present study confirm the presence of C5aR but not of C5L2 in the rabbit retina. C5aR expression in the retina is demonstrated before: one report detected the presence of C5aR in the inner plexiform layer and occasionally in the ganglion cell layer in human retina^23^, and another report showed that C5aR was expressed in the ganglion cell layer of mouse retina^24^. There are two kinds of cells in the ganglion cell layers: RGCs and DACs. Neither of the two reports could distinguish whether only one of them expresses C5aR. Finally, another report showed that retinal Müller cells could also express C5aR (*in vitro*)^25^. In the present study, only RGCs expressed C5aR. As PVL concentrates in RGCs and RGCs express C5aR (Fig. 3), we believe that PVL colocalizes with RGCs through C5aR.

DACs did not express C5aR, but they were transiently colocalized with PVL. Although it was shown that LukS-PV is unable to bind any blood cells without C5aR, all these studies investigated myeloid cells only^9^. The sensitivity of neurons to PVL was first addressed by Jover *et al*., who demonstrated the neurotoxic activity of HlgC/B and PVL^13^. Given that DACs are neurons, we cannot exclude the possibility of another PVL-binding mechanism

PVL concentration increased in RGCs with time, while PVL expression decreased in DACs within a few hours. The diverse kinetics of PVL association with RGCs and DACs is difficult to explain apart from another specificity for an eventual second receptor. RGCs are long-projection neurons and may establish links with other cells such as DACS and microglia or other glial cells. They send visual information through their long axons from the retina to the brain^26^. DACs are integrated interneurons without axons. Because they are located at the second synaptic level of light pathways, consisting of the photoreceptor-bipolar-ganglion cell chain, DACs play the role of modulating and interposing the signal transmitter^27^. Compared to RGCs, DACs are more resistant to neurodegeneration than RGCs after glaucoma and complete optic nerve transection^28^. Some selective RGCs may be postsynaptic to DACs^29^. However, little is known about the other relationships between RGCs and DACs.

Laventie *et al*. injected PVL and antibodies against S or/and F component in rabbit vitreous for 24 h^29^. The animal groups with humanised antibodies against either S or F component along PVL injection did not show a significant ocular inflammation, while the group injected with only PVL showed a great ocular inflammation. In this work, it was demonstrated that both the S and F components of leukotoxins were necessary to cause a physiological response^29^. Through the possible C5aR binding, PVL could initiate the rise of intracellular Ca^2+^ concentration and the release of glutamate, as was recently shown in newborn rat cerebellar granular neurons^13^. The rise of Ca^2+^ concentration can activate some signal pathways to produce pro-inflammatory cytokines, chemotaxis or neurotransmitters in neurons and initiate inflammation^30–32^. Chiu *et al*. showed that bacteria secrete N-formylated peptides and α-hemolysin, which directly induce calcium flux and action potentials through nociceptors at the end of sensory neurons^33^, resulting in the release of neuropeptides and neurogenic inflammation^34^.

The morphological changes in Müller and microglial cells were observed in this study at 30 min and 2 h respective after PVL injection. We found elevated inflammatory markers at 4 h post-PVL injection. It is difficult to deduce whether the activated glial cells are a consequence or a cause of neural dysfunction. Indeed, when the neuron system is subjected to injury due to inflammation or trauma the glial cells are activated and exhibit gliosis^18^. Activated Müller cells can disturb the structural support or metabolic function of neurons, resulting in their dysfunction and loss. The activated microglia can also modulate the expression of trophic factors by Müller cells, which indirectly affects photoreceptors^35,36^. However, in this study, we could not detect any neuronal damage at least until 8 h after PVL injection.

Nitrotyrosine represents reactive oxygen species and reactive nitrogen species, which have diffused in the retinal tissue^37^. Increased nitrotyrosine concentration reflects an underlying inflammatory process with significant NO production, and this was observed in the retinal tissue as early as 4 h after PVL injection. It has established that NO can downregulate the tight junction proteins occludin and ZO-1, resulting in the breakdown of the BRB^38^. The state of microglial cell activation is conversely correlated with cell viability. In a recent study, apoptosis of *in vitro* activated microglial cells was promoted by NO production. Indeed, microglial cells can produce NO and undergo apoptotic death when they are significantly activated^39^. As our study showed that some microglial cells underwent apoptotic death and nitrotyrosine concentration increased 4 h after PVL injection, there might be a significant correlation between NO production, microglial cell apoptosis, and retinal inflammation during PVL infection.

IL-6 interacts with its receptor and then elicits JAK/STAT (Janus kinase/signal transducer and activator of transcription) and MAPK (mitogen-activated protein kinase) pathways which enhance numerous biological activities^40^. IL-6 was proved to be associated to many ocular pathologies related to inflammations, such as uveitis, glaucoma, ocular neovascularization and autoimmune disease^16^. The humanized anti-human IL-6R mAb, Tocilizumab, is effective to treat refractory uveitis and potent new therapeutic for other ocular diseases^41^. Rojas *et al*. proved that intravitreal injection of angiotensin II caused increases of IL-6 mRNA and protein. IL-6 was localized to retinal microglia and/or macrophages^42^. The present study showed that IL-6 mRNA and protein in retina increased after 4 h and 8 h PVL injection. The increased IL-6 expression might be due to activated microglial cells and may play a great role in nitrotyrosine production and retinal inflammation.

Nevertheless, early activation of Müller and microglial cells may cause major consequences to the visual outcomes in this PVL-mediated endophthalmitis model^14^. Further investigations of the effects of PVL binding on neural activity and the relation between neurogenic inflammation and glial cell activation should be performed. Retinal explants and *in situ* sophisticated approaches might bring more insights about the sequential activity of retinal cells.

## Materials and Methods

### Animal and surgical procedure

The animal experiments were approved by the Ministère de l’Education nationale, de l’Enseignement supérieur et de la Recherche, France (APAFiS no. 4986). The surgical procedure was performed in accordance with the guidelines in the laboratory of the Association for Research in Vision and Ophthalmology within the accredited A67-482-34 and B67-482-34 animal facilities. Nine pigmented rabbits (Bleu de Champagne) aged one year and weighing 3.5–4 kg were anesthetized by a lumbar intramuscular injection of ketamine, 20 mg/kg (Virbac, Carros, France) and xylazine, 3 mg/kg (Bayer Healthcare, Puteaux, France). After local anesthesia of the eyeball with 2–3 drops of oxybuprocaine chlorhydrate (Théa, Clermont-Ferrand, France), PVL in phosphate-buffered saline (PBS) (3 µg/50 µL), purified from ATCC49775 *S*. *aureus*^43^, was intravitreally injected with a 30-Gauge needle inserted 4 mm behind the corneal limbus. For controls, three eyes were injected with 50-µL PBS using the same technique. After PVL injection, animals were sacrificed at 30 min, 1, 2 and 4 h (two animals: three eyes with PVL and one control eye in each group) first using anesthesia with ketamine-xylazine (as mentioned above), followed by a lethal intravenous injection of 2-mL Pentobarbital Dolethal^®^ (Vetoquinol, Lure, France) through a 22-Gauge catheter inserted in the marginal auricular vein.

### Eye preparation

The eyes were intravitreally injected with 100 μL of 4% (wt/vol) paraformaldehyde (Thermo Fisher Scientific, Rockford, IL, USA) immediately after sacrifice, and were then oriented and enucleated. The cornea, iris, and crystalline lens were immediately removed. Half of the eye globe was fixed for 3 h in 4% paraformaldehyde, and the remainder was immediately frozen. The dissected eyes were successively immersed in 10% (wt/vol) and 20% (wt/vol) sucrose and stored in 30% (wt/vol) sucrose overnight at 4 °C. For a better cryosection, the retina was separated from the pigmented epithelium. The temporal zone of 1–5 mm near the optic disc was isolated and then immersed in optimal cutting temperature compound (Sakura Finetek, Torrance, CA, USA). Vertical cryostat 8-µm-thick sections were mounted on a Super Frost^TM^ Plus microscope slides (Thermo Fisher Scientific, Rockford, IL, USA) and stored at −20 °C.

### Immunohistochemistry

Immunohistochemistry was performed to analyze the retinal cells targeted by PVL. PVL protein was stained using anti-LukS-PV-specific antibody. Retinal sections and whole mounts were permeabilized in 0.05% (v/v) and 0.1% (v/v) TritonX-100, respectively, for 1 h and then were blocked with 10% (v/v) donkey or goat serum (Sigma-Aldrich, St. Louis, MO, USA) for 1 h. Retinal sections were incubated with primary antibody (see Table 1 for details) at 4 °C overnight in a humidity chamber. Retinal sections were then incubated for 1 h at room temperature with fluorescent secondary antibodies or TUNEL (except when lectin was used) (see Table 1 for details). The sections were counter-stained with Hoechst 33258 and mounted in 10% (v/v) Mowiol^®^ solution (Polysciences, Eppelheim, Germany). Images of fluorescent sections and whole mounts were obtained using an epifluorescence Olympus BX60 microscope connected to a Hamamatsu C11440 digital camera.

### Cell counting

Five different microscope fields (266 µm × 266 µm) of vertical retinal sections were captured by the camera. The proportions of PVL-positive RGCs and DACs were measured in the RGC layer. PVL-positive cells were double-labeled by PVL and cell-specific markers for RGCs and DACs (Figs 1 and 2). The percentages of PVL-positive RGCs and DACs in each eye were established after triplicate experiments. For TUNEL positive cells count, five different microscope fields were analyzed for each retina.

#### Western blotting

One half of whole frozen retinas were homogenized by passing through a 26-Gauge needle several times in RIPA buffer [1% (v/v) NP-40, 0.1% SDS, 1% (w/v) sodium deoxycholate, 50 mM sodium chloride, 25 mM Tris-HCl pH 8.0] containing an inhibitor protease cocktail. The concentration of proteins in the supernatant was quantified using the BCA kit (660-nm Protein Assay Reagent, Pierce Biotechnology). The same amount of protein was loaded in each lane of SDS-PAGE gel (Bio-Rad Laboratories, Hercules, CA, USA). After migration, proteins were transferred to a nitrocellulose membrane, which was blocked in 5% (w/v) skimmed milk in PBS and probed in primary antibody anti-nitrotyrosine, anti-IL-6, anti-IL-8, anti-TNFα, anti-IL-1β (see Table 1) overnight at 4 °C. The membranes were washed three times in phosphate-buffered saline with Tween® (PBST) and incubated in Peroxidase-conjugated anti-mouse or anti-rabbit secondary antibodies (see Table 1) for 1 h at room temperature. The digital images were developed using ECL Western blotting detection reagent (Bio-Rad Laboratories, Hercules, CA, USA) and a chemiluminescence camera (ChemiDoc™ XRS, Bio-Rad). The protein expressions were quantified by densitometry analysis of Western Blots bands using BIO-1D software. The tests were triplicate.

### Real-time RT-qPCR

We analyzed tested retinas (PVL 4 h and PVL 8 h, 3 eyes for each group) and control retinas (PBS 4 h, 6 eyes) using RT-qPCR to see the elevation of cytokines as sign of retinal inflammation. The eyes were immediately dissected after the death of rabbit at the end of times course and put into CO_2_-independent medium (Gibco, Life technologies, Carlsbad, USA). The retinas were immediately isolated in CO2-independent medium and stocked immediately at −80 °C. Trizol reagent (Sigma, Saint-Louis, USA) was added into tube contained frozen retina, the retinas were passed through 23-Gauge needle then 26-Gauge needle several times to homogenize the retina. Total RNA was isolated using Trizol reagent according to the manufacturer’s instructions. The final RNA solutions were quantified with spectrophotometry (NanoDrop; Thermo Scientific, Waltham, USA). Then, 10 µg RNA aliquotes were treated with DNA-free kit DNase treatment (Ambion, Life technologies) at 37 °C for 30 min and removal reagents according to manufacturer’s instructions. RNA integrity was analyzed using non-denaturing agarose gel electrophoresis. Total RNA was immediately reverse transcribed(RT) using Superscript First-Strand Synthesis for RT-PCR (Invitrogen, Life technologies). Diethyl pyrocarbonatedecarbonate (DEPC) (Sigma) treated H_2_O was added to RT mixture (0.5 µl random hexamers (200 ng/ml), 500ng total RNA, 1 µl NTP) to achieve a 12 µl volume, then incubated at +65 °C for 5 min and placed in glass for 2 min. Then 0.5 µl of 0.1 M DDT, 0.5 μl of transcriptase, 4 µl of First Strand buffer, 3 ul of sterile H_2_O were added to the mixture. Then, the total mixture was put into ThermoCycler programmed at +42 °C for 50 min and at +70 °C for 15 min. The cDNA was diluted in 3 times with DEPC treated H_2_O. 5 µl of diluted DNA, 10 µl SYBR mix (LightCycler 480 SYBR Green I Master, Roche, Basel, Switzerland), 2 µl of forward and reverse primers, and 3ul H_2_O were mixed and put into 96 wells plate. The plate was placed into Real-Time PCR System (Light Cycler 480, Roche). The primers were designed to have Tm around 60 °C by using Primer3 software. PCR was programmed as initial denaturation step at +95 °C for 10 min, 45 cycles of amplification (denaturation at +95 °C for 15 s, annealing at +60 °C for 20 s, extension at +72 °C for 15 s), and melting curve analysis (+60 °C to +95 °C increment at +0.3 °C). The specificity of PCR products was verified according to one melting curve peak and one band in agarose gel electrophoresis. RTs without reverse transcriptase were used as controls to assure no significant DNA contamination. The sequences of primers: β-actin forward primer 5′-gcgggacatcaaggagaag-3′, afterward primer 5′-aggaaggagggctggaaga-3′; IL-6 forward primer 5′-tcaggccaagttcaggagtg-3′, afterward primer 5′-atgaagtggatcgtggtcgt-3′; IL-8 forward primer 5′-tggctgtggctctcttgg-3′, afterward primer 5′-atttgggatggaaaggtgtg-3′; TNFα forward primer 5′-cgtagtagcaaacccgcaag-3′, afterward primer 5′-tgagtgaggagcacgtagga-3′; IL-1β forward primer 5′-ttgtcagtcgttgtggctct-3′, afterward primer 5′-ggatttctgttgtgcatcct-3′; VEGF forward primer 5′-cgagaccttggtggacatctt-3′, afterward primer 5′-tgcattcacatttgttgtgct-3′; MCP-1 forward primer 5′-aacgcttctgtgcctgct-3′, afterward primer 5′-ggacccacttctgcttgg-3′. The β-actin was used as reference gene and target genes were normalized using this reference gene. The method ∆Ct was used to calculate relative quantification between control retina and tested retina. The fold changes were calculated using 2^−∆∆Ct^. The tests were triplicate. The significant changes of every target gene were statistically analyzed using ∆Ct paired t-tests.

### Statistical analysis

Statistical analysis was performed with GraphPad InStat version 3.10. Statistical significance was calculated with one-way ANOVA using the Tukey-Kramer multiple comparisons test and paired t-tests. Statistical significance was assumed at *p* < 0.05.

### Data availability

All data sets generated, including those that were analyzed during the current study, are available with the corresponding author on request.

## Electronic supplementary material

Supplementary figures
