# Supplementary material for: Panton–Valentine Leukocidin Colocalizes with Retinal Ganglion and Amacrine Cells and Activates Glial Reactions and Microglial Apoptosis
Source: Sci Rep. 2018 Feb 13;8:2953. doi: 10.1038/s41598-018-20590-z (PMC5811455; doi:10.1038/s41598-018-20590-z)
Supplement: Supplementary file 1 — Supplementary figures [file 41598_2018_20590_MOESM1_ESM.pdf]

Supplementary Figures S1-S7

Title: Panton–Valentine Leukocidin Colocalizes with Retinal Ganglion and Amacrine Cells and Activates Glial Reactions and Microglial Apoptosis

XuanLi LIU [1], Pauline HEITZ [2], Michel J[3]ROUX, Daniel KELLER [1], Arnaud SAUER [2], Gilles PREVOST [1], David GAUCHER [1,2#]

1. Université de Strasbourg, Hôpitaux Universitaires de Strasbourg, Fédération de Médecine Translationnelle de Strasbourg, EA7290 Virulence Bactérienne Précoce, Institut de Bactériologie, Strasbourg, France.

2. Hôpitaux Universitaires de Strasbourg, Service d'Ophtalmologie du Nouvel Hôpital Civil, Strasbourg Cedex - France.

3. Department of Translational Medicine and Neurogenetics, Institut de Génétique et de Biologie Moléculaire et Cellulaire, CNRS UMR_7104, Inserm U 964, Université de Strasbourg, Illkirch, France

The fax, telephone number, and e-mail address of the corresponding author:

Tel: +33 (0)3 69 55 11 15; +33 (0)6 63 12 75 98. Fax: +33 (0)3 69 55 18 49. Email: david.gaucher@chru-strasbourg.fr

Supplementary Figure S1.

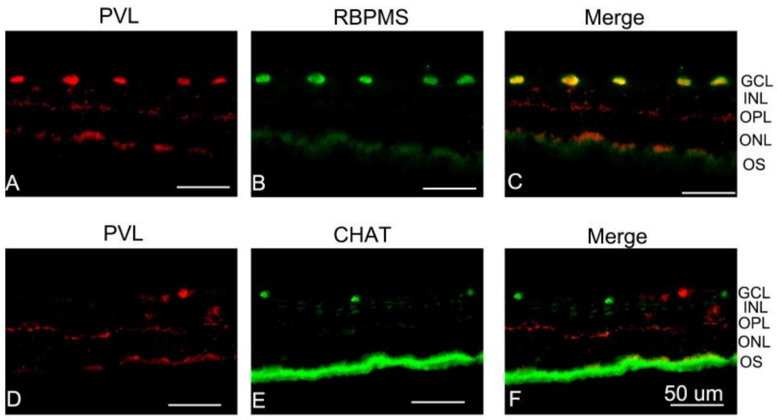

Supplementary Figure S1. PVL colocalized with RGCs and few DACs 8 h after PVL injection. PVL (red fluorescence A, C) colocalized with RGCs labeled with anti-RBPMS antibody (green fluorescence B, C) in the retinal vertical sections. DACs labeled with anti-CHAT antibody (green fluorescence E, F) did not colocalize with PVL (red fluorescence D, F).

Abbreviated symbols: RGCs, retinal ganglion cells; DACs, displaced amacrine cells; CHAT, choline acetyl transferase; RBPM, RNA-binding protein with multiple splicing; GCL, ganglion cell layer; OPL, outer plexiform layer; INL, inner nuclear layer; ONL, outer nuclear layer. OS, photoreceptor outer segments.

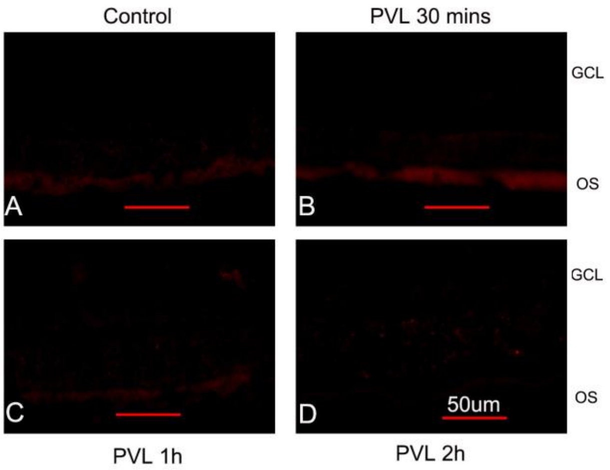

Supplementary Figure S2. TUNEL test were negative in controls and retinas 30 mins, 1 h, 2 h after PVL injection. The TUNEL test did not show any specific positive fluorescence in retina control(A), in retina 30 mins after PVL injection (B), in retina 1 h after PVL injection (C) and in retina 2 h after PVL injection (D).

Abbreviated symbols: TUNEL, terminal deoxynucleotidyl transferase dUTP nick-end labeling; GCL, ganglion cell layer; OS, photoreceptor outer segments.

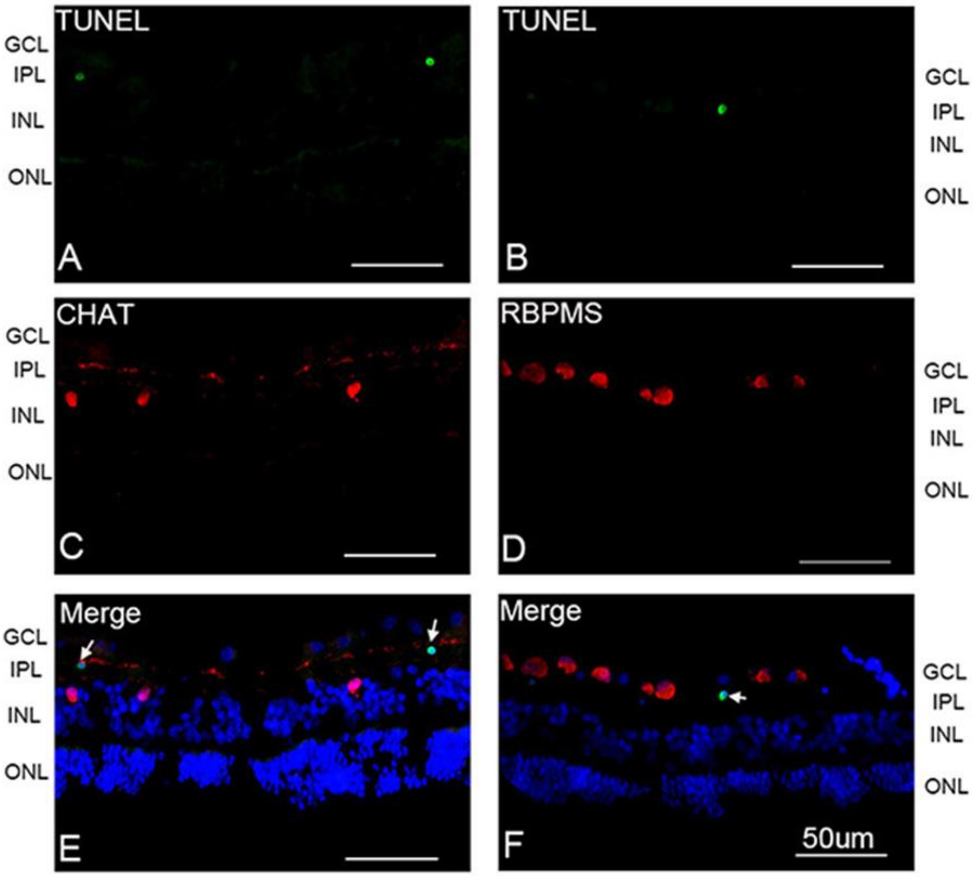

Supplementary Figure S3. RGCs and DACs did not colocalize with TUNEL-positive cells. The TUNEL-Positive cells (green fluorescence A, B, E, F) did not colocalize with anti-CHAT labeled DACs (red fluorescence C, E), nor with anti-RBPMS labeled RGCs (red fluorescence D, F). Hoechst stained nuclei (blue fluorescence E, F).

Abbreviated symbols: RGCs, retinal ganglion cells; DACs, displaced amacrine cells; TUNEL, terminal deoxynucleotidyl transferase dUTP nick-end labeling; CHAT, choline acetyl transferase; RBPM, RNA-binding protein with multiple splicing; GCL, ganglion cell layer; IPL, inner plexiform layer; INL, inner nuclear layer; ONL, outer nuclear layer.

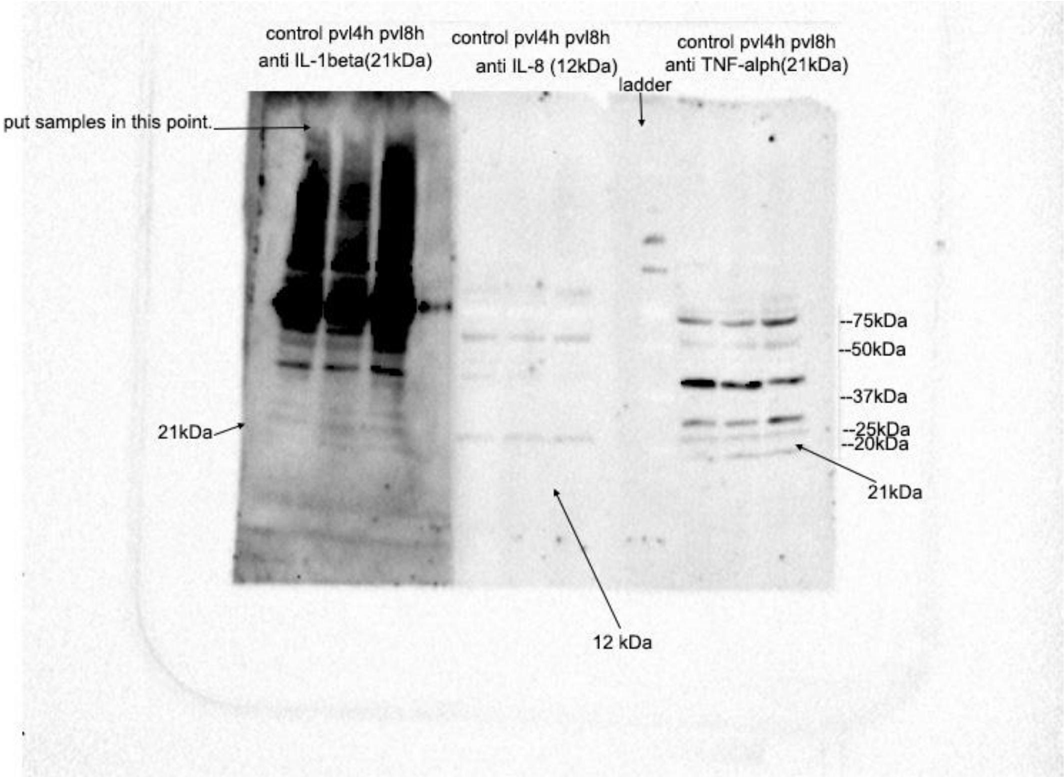

Supplementary Figure S4. The full-length blots for IL-1β, IL-8 and TNF-α. The primary antibodies were rabbit anti human IL-1β, mouse anti rabbit IL-8 and mouse anti-rabbit TNF-α from left to right. The lanes were put the same quantity proteins (40 ug/ lane) extracted form retina control, PVL 4 h, PVL 8 h from left to right according to the results of BCA kit. The specific bands for IL-1β were around the 21kDa, those for IL-8 were around 12kDa and those for TNF-α were around 21kDa. We could see that thses three factors have no sepecific bands in lanes of all samples (control, PVL 4 h, PVL 8 h) using western blotting method.

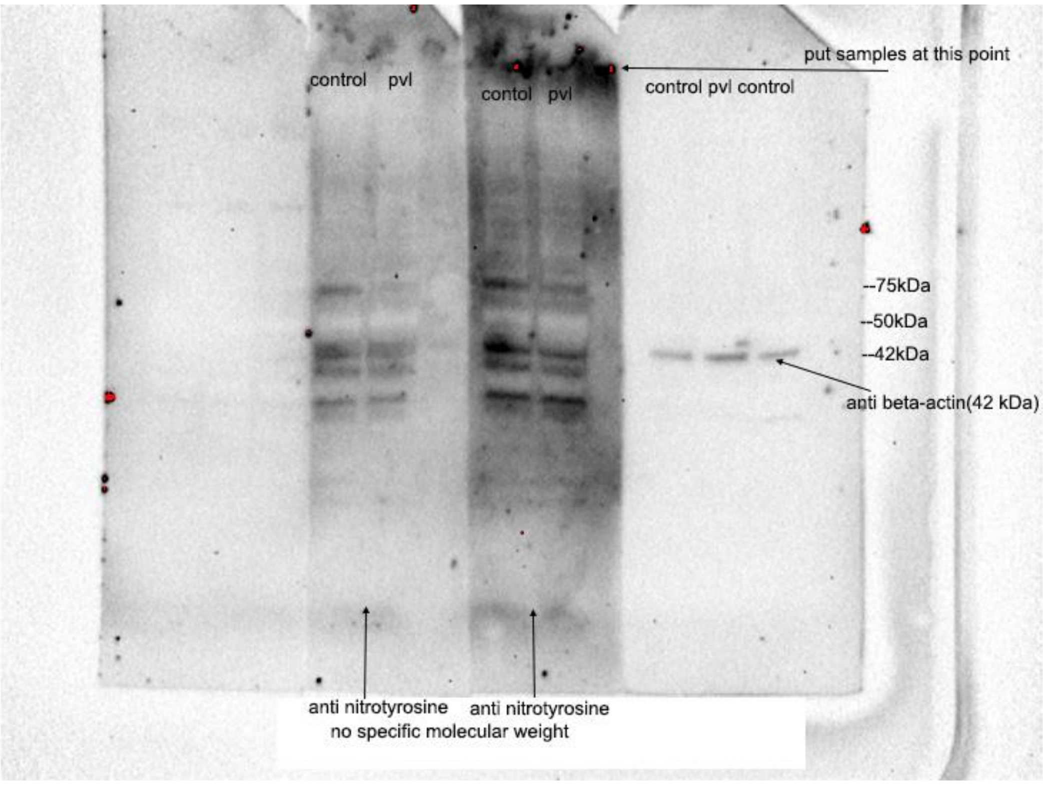

Supplementary Figure S5. The full-length blots for nitrotyrosine and β-actin. The first anti nitrotyrosine was treated with more detergents than the second anti nitrotyrosine. In this gel, all the lanes were loaded with the same quantity of test proteins (pvl 4h) and the same quantity of control proteins (40ug/lane) calculated with BCA kits. The anti-nitrotyrosine antibody recognizes free/protein-bound 3-NT which has no relation with molecular weight. The different bands correspond to different nitrotyrosine bound proteins that have between 37 kDa and 75 kDa molecular weight. The specific β-actin bands were showed around 42 kDa. The bots for nitrotyrosine and β-actin were from the same samples.

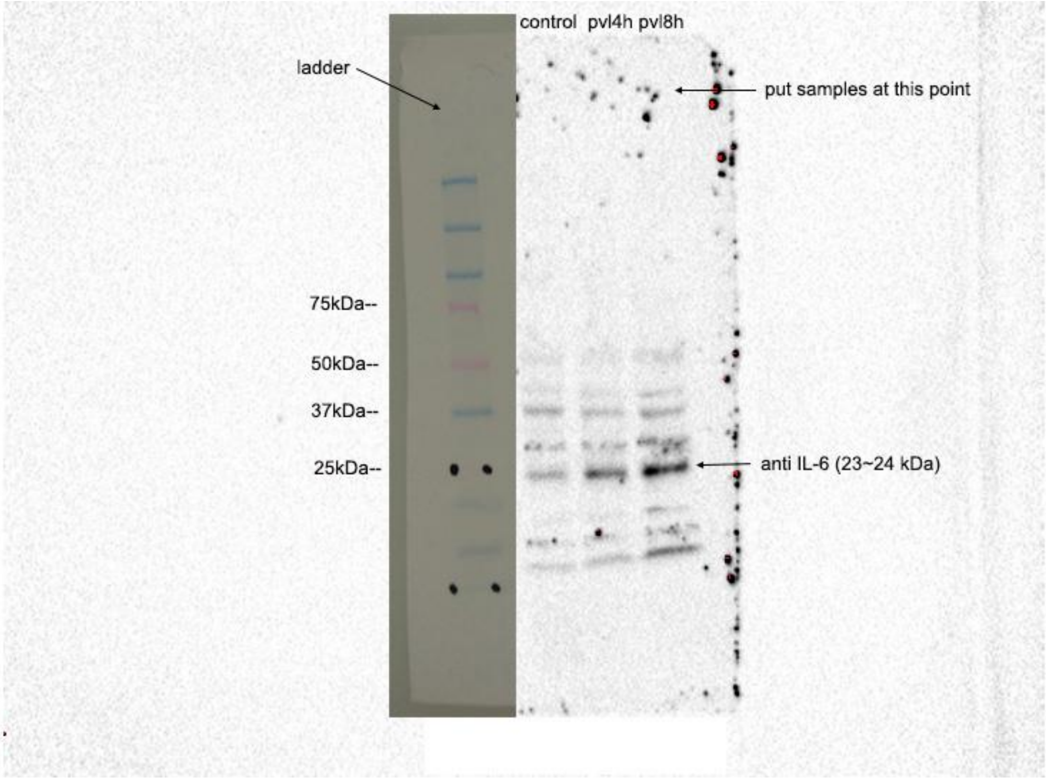

Supplementary Figure S6. The full-length blots for IL-6. The primary antibody was mouse anti human IL-6. The lanes were put the same quantity proteins (40 ug/ lane) extracted form retinas (control, PVL 4 h, PVL 8 h from left to right) according to the results of BCA kit. The specific bands were around the 24kDa. We could see clear bands in lanes around 24kDa of PVL 4 h and PVL 8 h, weak band around 24kDa in lane of control.

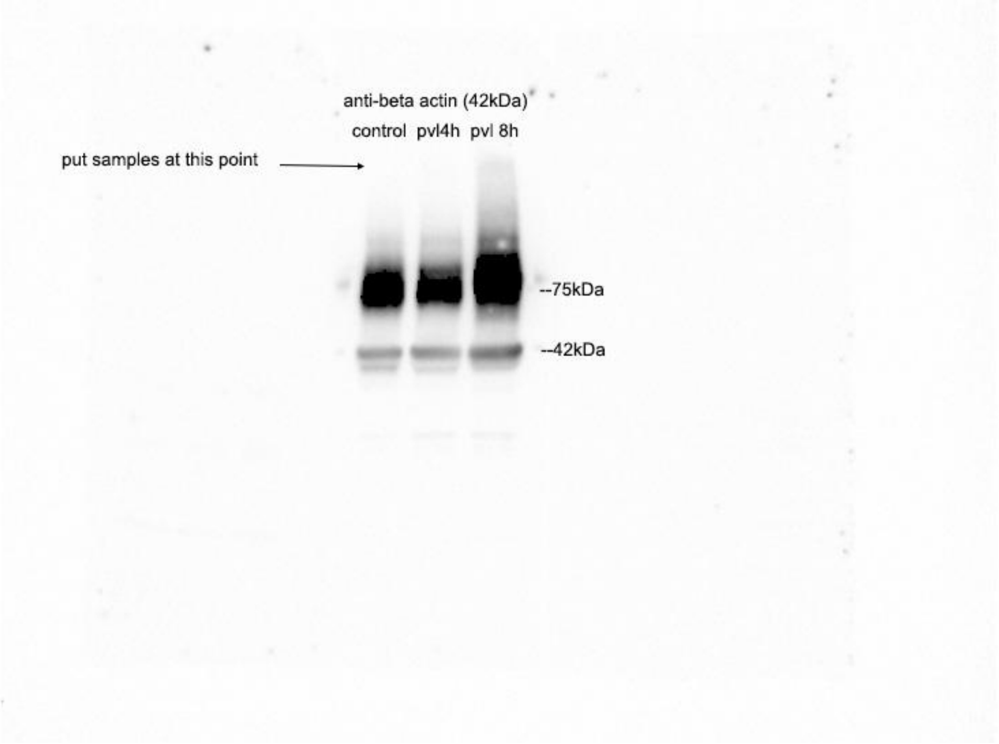

Supplementary Figure S7. The full-length blots for β-actin with IL-6 The primary antibody was rabbit anti human β-actin, the lanes were put the same quantity proteins (40 ug/ lane) extracted form retinas (control, PVL 4 h, PVL 8 h from left to right) according to the results of BCA kit. The specific bands were around the 42kDa.
